# Biological Investigation and Chemical Study of *Brassica villosa* subsp. *drepanensis* (Brassicaeae) Leaves

**DOI:** 10.3390/molecules27238447

**Published:** 2022-12-02

**Authors:** Giuseppe Antonio Malfa, Marinella De Leo, Rosa Tundis, Alessandra Braca, Monica Rosa Loizzo, Claudia Di Giacomo, Francesco Maria Raimondo, Anahi Elena Ada Bucchini, Rosaria Acquaviva

**Affiliations:** 1 Department of Drug and Health Science, University of Catania, Viale A. Doria 6, 95125 Catania, Italy; 2 Research Centre on Nutraceuticals and Health Products (CERNUT), University of Catania, Viale A. Doria 6, 95125 Catania, Italy; 3 PLANTA/Autonomous Center for Research, Documentation and Training, Via Serraglio Vecchio 28, 90123 Palermo, Italy; 4 Department of Pharmacy, University of Pisa, Via Bonanno 33, 56126 Pisa, Italy; 5 Research Centre for Nutraceutical and Healthy Foods “NUTRAFOOD”, University of Pisa, Via del Borghetto 80, 56124 Pisa, Italy; 6 Centre for Instrumentation Sharing, University of Pisa (CISUP), Lungarno Pacinotti 43, 56126 Pisa, Italy; 7 Department of Pharmacy, Health and Nutritional Sciences, University of Calabria, Via P. Bucci, 87036 Rende, Italy; 8 Department of Biomolecular Sciences, Center of Research ‘Botanic Garden’, University of Urbino, Via Bramante 28, 61029 Urbino, Italy

**Keywords:** wild cabbages, *Brassica villosa* subsp. *drepanensis* (Caruel) Raimondo & Mazzola, polyphenols, glucosinolates, cancer cells, inflammation, LPS, NO, ROS, LC-MS analysis

## Abstract

*Brassica villosa* subsp. *drepanensis* (Caruel) Raimondo & Mazzola, belonging to the *Brassica oleracea* complex, is a wild edible plant endemic to western Sicily and a relative of modern cultivated *Brassica* crops. In this study, the antioxidant properties, anti-inflammatory activities, enzymatic inhibition, and cytotoxicity in cancer cells of *B. villosa* subsp. *drepanensis* leaf ethanolic extract were analysed for the first time. In addition, its chemical profile was investigated partitioning the total 70% ethanol extract among ethyl acetate, *n*-butanol, and water to obtain three residues that were subjected to chromatographic separation. Two flavonol glycosides, a phenol glucoside, two amino acids, and purine/pyrimidine bases were obtained. The presence of the glucosinolate glucoiberin was detected in the water extract by UHPLC-MS analysis. The total polyphenol and flavonoid content of the 70% ethanol extract showed good antioxidant capacities and anti-inflammatory properties by reducing nitric oxide release and reactive oxygen species levels and increasing glutathione in lipopolysaccharide-stimulated RAW 264.7 cells. The extract inhibited the enzymatic activity of α-amylase, α-glucosidase, and, significantly, of lipase. The MTT assay showed that the extract did not affect the viability of normal HFF-1 and RAW 264.7 cells. Among the cancer cell lines tested, an antiproliferative action was only observed in CaCo-2. The cytotoxicity of the extract was further confirmed by LDH release assay and by the destabilization of the oxidative balance. Results confirmed the antioxidant properties of the crude extract responsible for the anti-inflammatory effect on healthy cells and cytotoxicity in cancer cells.

## 1. Introduction

Since immemorial time, wild edible plants have represented the primary source of food and subsistence for humans. Some of them are genetically related to cultivated crops and possess substantial potential both for the development of new crops through domestication and for interspecific hybridization in the selection of new and improved varieties in terms of tolerance to abiotic stresses, pests, disease resistance, and, last but not least, better bioactive secondary metabolite content [1,2]. It is well-known that wild relatives of edible plants show a different and richer phytochemical profile compared to the more cultivated varieties [3]. Many studies in the last decades have focused on edible plants such as *Brassica* spp., capable of effectively fighting against the development of numerous diseases and presumably able to prevent their onset [4]. It is widely demonstrated that a chronic inflammatory state is a transversal factor in many modern pathologies with a high social and economic impact [5]. Beside the fact that inflammatory responses are fundamental for the restoration of normal physiological functions following a pathological event, recent research has revealed that some social, environmental, and lifestyle factors can promote a state of chronic systemic inflammation that can in turn lead to the genesis of several pathologies known as lifestyle diseases, representing the main causes of disability and mortality around the world [6,7]. Macronutrients and micronutrients introduced into the diet are essential for the maintenance and function of immune cells. In fact, nutrient deficiencies negatively affect the immune system [8]. On the other hand, a dietary model rich in micronutrients with anti-inflammatory properties and low in pro-inflammatory nutrients can protect against diseases related to a chronic inflammatory state, including metabolic disorders and cancers [9]. Furthermore, health-promoting agents such as phenol compounds and glucosinolates, typical constituents of *Brassica* vegetables, have been shown to be involved in many biological functions with beneficial effects on human health [10,11]. *Brassica villosa* subsp. *drepanensis* (Caruel) Raimondo & Mazzola (Brassicaceae) (*B. drepanensis*) is an edible plant endemic to Sicily, only present in restricted areas between Trapani and Palermo and growing in coastal and rocky environments at altitudes varying from 0 to 600 m [12]. This plant has been poorly studied, and in the past, was the subject of genetic improvement programs for cultivated *Brassica oleracea* L. species, as it shows a higher content of total phytochemical compounds [13].

Therefore, the aim of this research was the phytochemical study of *B. drepanensis* leaves and, for the first time, the evaluation of their anti-inflammatory and antioxidant activities on the murine macrophage RAW 264.7 as an in vitro model of lipopolysaccharide (LPS)-induced inflammation. In addition, the inhibitory effects on digestive enzymes relevant to carbohydrate and lipid metabolism and cytotoxicity on human HFF-1 fibroblasts, healthy murine RAW 264.7 cells, and on four tumour cancer cell lines were evaluated.

## 2. Results

### 2.1. Phytochemical Analysis

The dried leaves of *B. drepanensis* were first extracted with EtOH-H_2_O 70% (*v/v*), then the extract was partitioned among EtOAc, *n*-BuOH, and H_2_O, obtaining the respective residues, and further subjected to a purification process through different chromatographic techniques to obtain pure compounds. The fractionation of the *n*-BuOH residue by reverse-phase high-performance liquid chromatography (RP-HPLC) afforded the isolation of five pure substances, identified as phenylalanine (**1**) [14], tryptophan (**2**) [14], roseoside (**3**) [15], sinapoyl glucoside (**4**) [16], and kaempferol 3-*O*-2”-sinapoylsophoroside-7-*O*-β-d-glucopyranoside (**5**) [17]. The aqueous residue was first fractionated by gel filtration column and the obtained fractions purified by RP-HPLC, leading to another three pure compounds, identified as uridine (**6**) [18], kaempferol 3-*O*-sophoroside-7-*O*-β-d-glucopyranoside (**7**) [17], and adenine (**8**) [19]. No specialized metabolites were obtained by the fractionation of the EtOAc extract using flash silica gel column chromatography followed by RP-HPLC. The structure of all compounds was established based on 1D and 2D NMR experiments, as well as electrospray ionization source (ESI)-tandem mass spectrometry (MS/MS) analyses.

**Figure 1:**
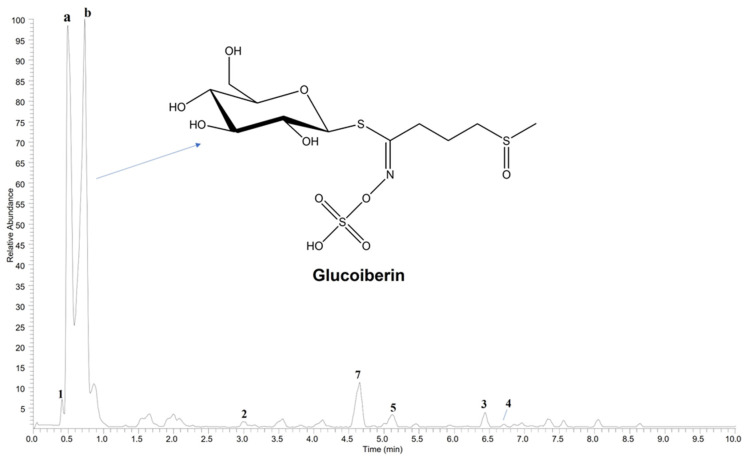
UHPLC-HR-MS profile of the aqueous fraction of *Brassica drepanensis* leaves, acquired in negative ion mode. **1** = phenylalanine; **2** = tryptophan; **3** = roseoside; **4** = sinapoyl glucoside; **5** = kaempferol 3-*O*-2”-sinapoylsophoroside-7-*O*-β-d-glucopyranoside; **7** = kaempferol 3-*O*-sophoroside-7-*O*-β-d-glucopyranoside; **a** and **b** = glucoiberin isomers.

**Figure 2:**
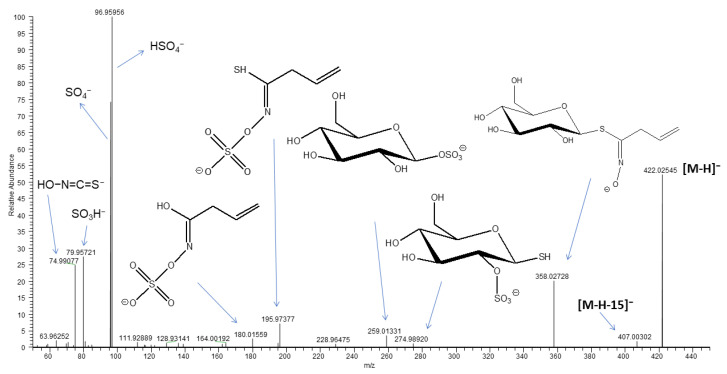
ESI-MS/MS of peaks **a** and **b** shown in Figure 1 and tentatively identified as glucoiberin.

During the fractionation process, no glucosinolates were isolated from the three investigated subfractions of the whole *B. drepanensis* leaf extract. Since glucosinolates are the major class of secondary metabolites expected in *Brassica* vegetables, all residues were deeply investigated using ultra-high performance liquid chromatography (UHPLC) coupled to a high-resolution mass spectrometry (HR-MS) instrument, a highly sensitive and accurate technique useful for characterizing constituents even in complex mixtures. Interestingly, the presence of glucosinolates was revealed only in the aqueous residue, together with other isolated compounds. The LC-MS profile of the aqueous fraction (Figure 1) confirmed the presence of compounds **1**–**7** according to the results obtained by the isolation process. Furthermore, peaks **a** and **b** were attributed to molecules not purified from any of the studied fractions. Based on MS fragmentation pathways, peaks **a** and **b** were assigned to the glucosinolate class. The full ESI-MS of both peaks acquired in negative ion mode showed a deprotonated molecular ion [M-H]^−^ at *m/z* 422.0255 (Figure 2) suggesting the occurrence of two isomers. In the ESI-MS/MS experiments, typical product ions of glucosinolates were observed at *m/z* 74.99 [S=C=N−OH]^−^, 79.96 [SO_3_H]^−^, 95.95 [SO_4_]^−^, and 96.96 [HSO_4_]^−^, due to the cleavage of the isothyocianate residue (Figure 2). In addition, product ions at *m/z* 274.99, 259.01 and 195.97, were generated by the loss of a thioglucose and the transfer of a sulfate group [20]. The product ion at *m/z* 358.03 was generated by the partial cleavage of the alkyl chain, while the product ion at *m/z* 407.00 by the loss of a methyl group ([M-H-15]^−^), probably located on the chain itself. These data are in agreement with the fragmentation pattern observed for 3-methylsulfinylpropyl glucosinolate (or ω-methylsulfinylpropyl glucosinolate), also known as glucoiberin, an alifatic glucosinolate previously reported in *Brassica oleracea* L. [21]. Thus, peaks **a** and **b** were tentatively assigned to isomeric forms of glucoiberin.

### 2.2. In Vitro Cell Free Antioxidant Assay

Antioxidant activity was assessed using different assays to better characterize the antioxidant power of the phytocomplex contained in the total 70% ethanol extract of *B. drepanenensis* leaves and in the obtained fractions (*n*-BuOH_,_ EtOAc_,_ and H_2_O). In Table 1 the results for 2,2’-azino-bis(3-ethylbenzothiazoline-6-sulphonic acid) diamonium salts (ABTS), 2,2-diphenyl-1-picrylhydrazyl (DPPH), superoxide dismutase-like activity (SOD-like activity), ferric reducing activity power (FRAP), and β-carotene bleaching tests are reported.

ABTS and DPPH tests were applied in order to investigate the radical-scavenging ability of *B. drepanensis*. The whole extract showed a promising activity in the ABTS test, with an IC_50_ value of 3.32 ± 0.88 μg/mL; conversely the DPPH test showed a dose-dependent quenching effect but with a high IC_50_ value of 368 ± 1.7 μg/mL. It is reported that the ABTS assay is better than the DPPH assay for evaluating the antioxidant power of a plant extract from a food matrix [22]. Afterward, the scavenger effect of the extract by SOD-like assay, using a method that excludes Fenton-type reactions and the xanthine/xanthine oxidase system, was tested. The whole extract was found to have a significant, dose-dependent scavenger effect, with an IC_50_ value of 66.66 ± 1.1 μg/mL, equivalent to 40 mU of SOD, demonstrating the good antioxidant activity of *B. drepanensis* extract not detected with the DPPH assay. This discrepancy is probably due to the smaller size of the O_2_^.^, which is more receptive to small molecules compared to the more sterically bulky DPPH radical. Lastly, the whole extract showed an appreciable reducing power, with an IC_50_ value of 10.46 ± 1.17 µMFe (II)/g (lower than the BHT used as a reference standard) but with a weak ability to contrast lipid peroxidation, as shown in the β-carotene bleaching test reporting IC_50_ values of 38.34 ± 2.51 and 47.95 ± 2.64 μg/mL after 30 and 60 min of incubation, respectively. Regarding the antioxidant properties of the three obtained fractions, the *n*-BuOH showed the best activity, even with respect to the total extract (Table 1). In contrast, the other two residues (EtOAc and H_2_O) showed fragile antioxidant properties in all the tests performed, with IC_50_ values remarkably higher than those evidenced for the whole extract and the *n*-BuOH fraction.

### 2.3. Cell Viability

**Figure 3:**
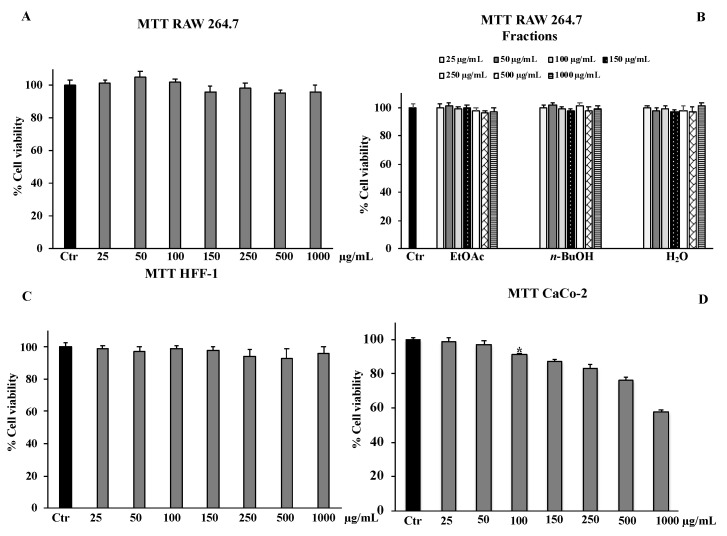
Cell viability in RAW 264.7 (**A**,**B**), HFF-1 (**C**), and CaCo-2 (**D**) cells untreated (Ctr) and treated for 24 h with the total extract or fractions (EtOAc, *n*-BuOH, or H_2_O). Values are the mean ± S.D. of five experiments in triplicate. Confidence intervals calculated by two-way ANOVA and Tukey post-hoc test: * Significant vs. untreated control cells: *p* < 0.001.

Results from MTT assays in healthy normal cells (HFF-1 and RAW 264.7) indicated that the crude extract at all concentrations tested (25–1000 μg/mL) had no effect on cell viability, as well as the three fractions obtained from the total extract and tested at the same concentrations (Figure 3A–C). The exposure of the tumour cells to the extract for 24 h produced an anti-proliferative effect only on the CaCo-2 cells (Figure 3D) (IC_50_ doxorubicin, 21 ± 0.3 μM), while no effect was shown on DU-145, 1321N1, and U87 MG cell lines at all tested concentrations (25–1000 μg/mL) (not relevant to be depicted in a figure).

Subsequently, the fractions were tested on CaCo-2 cells since they were responsive to the treatment with the total extract. No anti-proliferative activity was detected for the three fractions (not relevant to be depicted in a figure).

### 2.4. ROS Levels

**Figure 4:**
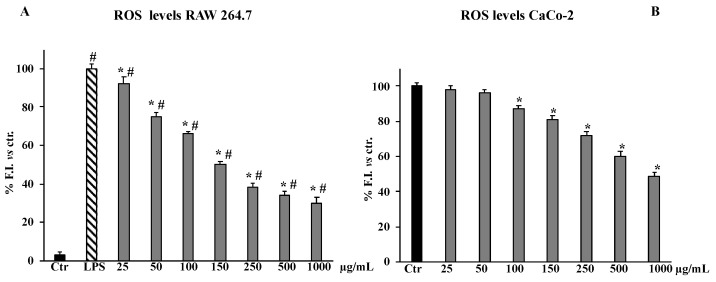
ROS levels in RAW 264.7 (**A**) and CaCo-2 (**B**) untreated cells (Ctr), LPS-treated for 2 h, and pre-treated with the total extract (25–1000 μg/mL) for 24 h. Values are the mean ± S.D. of five experiments in triplicate. Confidence intervals calculated by two-way ANOVA and Tukey post-hoc test: ^#^ Significant vs. untreated control cells: *p* < 0.001; * Significant vs. LPS-treated cells: *p* < 0.001.

ROS levels in RAW 264.7, increased by 100% LPS treatment respective to the control, were significantly reduced by pre-treatment with the total extract. Figure 4 shows that the total extract inhibited ROS production in a dose-dependent manner from 25 to 250 μg/mL with respect to the cell treated with LPS alone, while at the concentrations of 500 and 1000 μg/mL no dose-dependent effect was highlighted, although both concentrations were significant with respect to RAW 264.7 LPS-activated cells. No activity against ROS production was detected for the three fractions in the same cell line (data not shown), despite the *n*-BuOH fraction showing the best antioxidant properties in cell-free in vitro tests. This result is probably due to the presence of kaempferol 3-*O*-2”-sinapoylsophoroside-7-*O*-β-d-glucopyranoside in the *n*-BuOH fraction. A recent study by Wang et al. demonstrated that the best biological effects, including antioxidant activity, are correlated to the kaempferol aglycone rather than to its glycosides, for some of which weak or no activities were highlighted [23].

In addition to investigating the possible role of oxidative stress in the antiproliferative effect of *B. drepanensis* extract on CaCo-2 cells, ROS levels were also evaluated. Figure 4 shows that the total extract reduced ROS levels starting from 100 μg/mL in a dose-dependent manner, confirming that the extract in both examined cell lines acts as an antioxidant agent.

### 2.5. Total Thiol Groups

**Figure 5:**
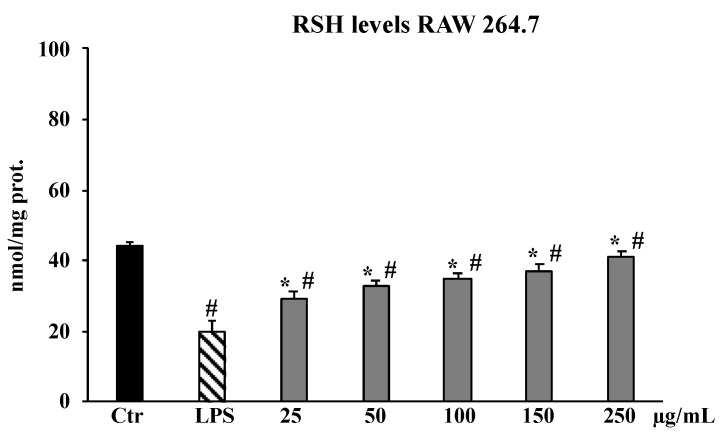
RSH in RAW 264.7 untreated cells: (Ctr), LPS-treated for 2 h, and pre-treated with the total extract (25–50–100–150–250 μg/mL) for 24 h. Values are the mean ± S.D. of five experiments in triplicate. Confidence intervals calculated by two-way ANOVA and Tukey post-hoc test: ^#^ Significant vs. untreated control cells: *p* < 0.001; * Significant vs. LPS-treated cells: *p* < 0.001.

Based on the inhibition trend of ROS levels observed in RAW 264.7 cells, we decided to narrow the concentrations of the crude extract for the next experiments (25–250 μg/mL) and to eliminate the fractions as no effect was up to now highlighted. The treatments with the total ethanolic extract of *B. drepanensis* resulted in a significant increase in total thiol group (RSH) levels with respect to LPS-treated cells where RSH content was depleted by the toxin. In particular, at 250 μg/mL RSH content was comparable to that reported for untreated control cells (Figure 5).

### 2.6. NO Release

**Figure 6:**
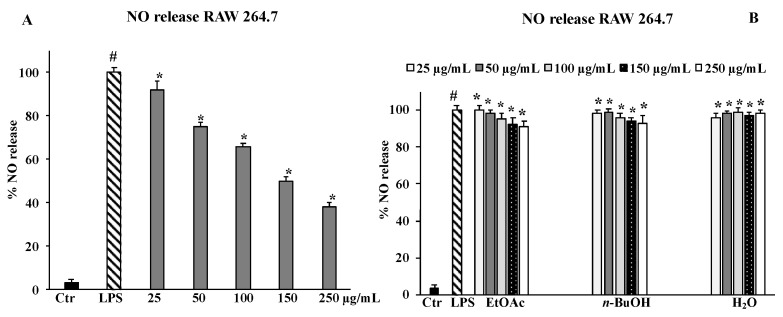
RAW 264.7 untreated cells (Ctr), LPS-treated for 2 h, and pre-treated with the total extract (25–50–100–150–250 μg/mL) (**A**) and fractions (EtOAc, *n*-BuOH, H_2_O) (**B**) for 24 h. Values are the mean ± S.D. of five experiments in triplicate. Confidence intervals calculated by two-way ANOVA and Tukey post-hoc test: ^#^ Significant vs. untreated control cells: *p* < 0.001; * Significant vs. LPS-treated cells: *p* < 0.001.

As shown in Figure 6A, the exposure to LPS increased NO· release in RAW 264.7 cells as expected, and the pre-treatment with *B. drepanensis* extract at all concentrations tested (25–250 μg/mL) was able to exert an inhibitory effect on NO· production in a dose-dependent manner. At the higher concentration (250 μg/mL), the total extract showed a decrease in the rate of radical NO· release of more than 50% compared to LPS-stimulated cells (Figure 6A). As for ROS production, the fractions did not significantly reduce NO· levels (Figure 6B). Based on these results, we decided to no longer investigate the three fractions but only the whole extract.

### 2.7. Enzymatic Inhibitory Activity Test

Results regarding the hypoglycaemic, hypolipidemic, and lipase activity of *B. drepanensis* whole extract are summarized in Table 2. The extract showed inhibition of α-amylase, α-glucosidase, and lipase in a concentration-dependent manner. The most promising activity was found against lipase enzymes, with an IC_50_ value of 74.84 ± 3.67 μg/mL, while a weak inhibition was evidenced for the carbohydrate-hydrolysing enzymes α-amylase and α-glucosidase.

### 2.8. Cytotoxicity in Cancer Cell Line

**Figure 7:**
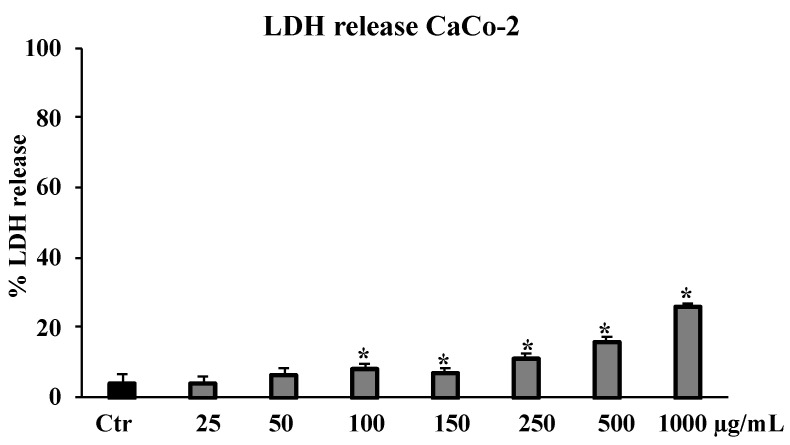
LDH release in untreated CaCo-2 cells (Ctr) and cells treated for 24 h with the total extract (25–1000 μg/mL). Values are the mean ± S.D. of five experiments in triplicate. Confidence intervals calculated by two-way ANOVA and Tukey post-hoc test: * Significant vs. untreated control cells: *p* < 0.001.

As shown in Figure 7, consistent with the results obtained by MTT assay on proliferation rate, the total extract induced cytotoxicity in the CaCo-2 cell line in a dose-dependent manner, as evidenced by an LDH release assay. The highest value, of about 40%, was reached at the highest tested concentration of 1000 μg/mL.

### 2.9. Total Phenol and Flavonoid Content

The total phenol content (TPC) of *B. drepanensis* whole extract was 29.87 ± 0.14 mg of gallic acid/g extract while the total flavonoid content (TFC) was 26.12 ± 0.09 mg of catechin/g extract (Table 3). The total phenolic content determined by the Folin–Ciocalteau method is comparable to that of other hydroalcoholic extracts obtained from other previously analysed species [24].

## 3. Discussion

Today, the health benefits of the Mediterranean diet are demonstrated by numerous scientific studies [25,26], which highlight that the beneficial effects on humans are ascribable (in addition to the nutrients that characterize it) to a variety of micronutrients and phytochemicals present in the plant-based foods belonging to Mediterranean native species such as olives, oats, barley, grapes, almonds, cabbages, and many species of herbs and aromatic plants [3,27,28]. Most of the health effects related to the Mediterranean diet are attributable to a reduction in a chronic inflammatory state and consequently to a reduction in the incidence of inflammation-related diseases, also known as non-communicable or lifestyle diseases, such as cardiovascular and neurodegenerative disease, diabetes, cancer, etc. [29]. Many in vitro and in vivo studies support the anti-inflammatory effect of phytochemicals from different *Brassica* spp. and their cultivated varieties [30]. *B. drepanensis* is a wild relative of cultivated cabbages. In this study, the total phenolic content of the ethanolic extract was determined for this taxon for the first time. The results obtained by the Folin–Ciocalteau method revealed an amount of phenolic compounds (29.87 ± 0.14 mg GAE/g) that is higher than other 70% ethanol extracts reported for different cultivated varieties of *B. oleracea* L. (min 4.30, max 13.80 mg GAE/g); nevertheless it is lower than those reported for two other wild taxa belonging to the *Brassica* genus, namely *B. incana* (37.20 mg GAE/g extract) and *B. fruticulosa* (32.63 mg GAE/g extract) [24,31].

Phytochemical investigation of the *B. drepanensis* hydroalcoholic extract evidenced that the leaves are a source of the amino acids phenylalanine and tryptophan (compounds **1** and **2**), as well as of phenol components (compounds **5** and **7**) having kaempferol as aglycone, glucose units in the saccharide chain, and a sinapoyl residue esterified with the sugar. These flavonol glycosides are common in *Brassica* plants [32]. Interestingly, both flavonoids **5** and **7** were isolated in good amounts (1.9 and 7.0 mg, respectively) from the leaves of *B. drepanensis*. Similarly, the isolation of sinapoyl glucose (compound **4**), is in agreement with the presence of hydroxycinnamic acid conjugated to saccharides, commonly found in other *Brassica* species [32]. The occurrence of the glucosinolate glucoiberin, detected and tentatively identified by LC-MS analysis, enriched the chemical profile of *B. drepanensis* leaves with a substance typical of the genus and of great significance to human health.

All the above-listed phytochemicals are ubiquitous secondary metabolites in *Brassicaceae* spp. with well-known antioxidant properties. Plant secondary metabolites show different antioxidant properties depending on the experimental model and the method of analysis [33]. In this study, to evaluate the antioxidant profile of the total extract, five in vitro cell-free assays were used. Results obtained by these different tests showed that *B. drepanensis* extract possess good dose-dependent primary antioxidant properties, as demonstrated by ABTS assay (IC_50_ 3.32 ± 0.88 µg/mL) and SOD-like activity assay (IC_50_ 66.66 ± 1.1 µg/mL); in addition, the FRAP assay value (IC_50_ 10.46 ± 1.17 µMFe/g) highlighted the high reducing power of the extract compared to the reference compound BHT (IC_50_ 63.24 ± 2.34 µMFe/g) (Table 1). Previous studies on *B. incana* and *B. fruticulosa* reported lower values for the same tests [24,31]. These differences are probably due to the compositions of the extract, which is influenced by plant phenology at the moment of harvesting and by differences in the applied extraction process [34].

A chronic inflammation state is the result of a continuous active inflammation response mediated by immune cells, including macrophages [35]. The sustained inflammatory process induces a condition of oxidative stress that reduces the antioxidant capacity of cells, causing cellular damage responsible for tissue homeostasis dysregulation and onset of related disorders [36]. Thus, the promising antioxidant and possible anti-inflammatory properties of the crude extract of *B. drepanensis* were further investigated in cells to better understand the antioxidant activities in a cellular system of oxidative stress/inflammation induced by LPS on RAW 264.7 cells [37]. We first assessed that the crude extract and its fractions did not affect cell viability, measured by the MTT test, of either murine RAW 264.7 or human HFF1 cells, the latter used as a control cell line (Figure 3A–C). Subsequently, the data obtained on antioxidant activity in a cellular model confirmed the extract as an excellent antioxidant agent. Pre-treatment with the *B. drepanensis* total extract on RAW264.7 cells counteracted the production of ROS (Figure 4A), and concomitantly preserved glutathione content depleted consequent to LPS exposure (Figure 5). Glutathione is one the most important non-enzymatic antioxidants in cells [38]. Depending on their functional groups and structure, polyphenols are powerful antioxidant agents due to their ability to scavenge free radicals and reduce their formation. Several investigations have shown that polyphenols, particularly the subclass of flavonoids considerably present in our extract, protect cells from oxidative damage with different mechanisms of action both in vitro and in vivo [39,40]. Macrophages play a pivotal role in most stages of inflammation, including chronic inflammation [35]. The release of nitric oxide (NO·) by infiltrating macrophages during the inflammation process mediates the inflammatory response in tissues. Inflammation caused by LPS in macrophages promotes NO· release, mediated by the inducible isoform of NO synthase [41]. We evaluated NO· production promoted by LPS exposure, demonstrating that *B. drepanensis* total extract significantly decreased NO production in a dose-dependent manner after 2 h of pre-treatment (about 50%) in RAW 264.7 cells (Figure 6A). Surprisingly, the *n*-BuOH fraction, which showed the best antioxidant properties with respect to the whole extract as well, did not show any difference at all the concentrations tested, nor did the two other fractions (Figure 6B). These results, together with the weak antioxidant properties of the other two fractions (EtOAc, H_2_O), suggested that the anti-inflammatory action is attributable not to a group of phytochemicals present in the extract, but rather to a synergistic action of all the components of the phytocomplex. A review has recently reported that, upon fractionation, plant extracts frequently lose their biological activities, suggesting that the exerted effects result from the whole phytocomplex, acting synergistically or additively, rather than a single fraction or compound [42]. Several studies reported that secondary metabolites from the Brassicaceae family, including kaempferol, possess valuable in vitro and in vivo anti-inflammatory and antioxidant activities [30], in agreement with our findings confirming the high potential of *B. drepanensis* extract in counteracting the early stages of oxidative stress and inflammation, which are transversals in many pathological conditions, including obesity and its associated complications, such as type 2 diabetes [29,43]. The effects of natural compounds on metabolic disorders differ, and include amelioration in energy expenditure, insulin secretion, glucose utilization, satiety, or beneficial interference with the gut microbiome [44]. For these reasons, *B. drepanensis* extract has been investigated as a potential agent to inhibit α-amylase, α-glucosidase, and lipase enzymes. The extract showed an ability to inhibit these enzyme activities in a concentration-dependent manner (Table 2). The most promising activity was found against lipase enzymes, with an IC_50_ value of 74.84 ± 3.67 μg/mL, while a weak inhibition was evidenced for the carbohydrate-hydrolysing enzymes α-amylase and α-glucosidase. Recently, Nallamuthu et al. investigated the anti-adipogenic effects of the alcoholic extract of *Brassica oleracea* on 3T3-L1 pre-adipocytes, as well as its lipase inhibitory properties [45]. The total extract showed a concentration-dependent lipase inhibitory activity (IC_50_ value of 74.2 μg/mL) and a non-competitive type of inhibition. Moreover, in a cellular system, *B. oleracea* extract inhibited intracellular triglyceride accumulation. The investigation of crude extracts and epidemiological studies showed that *Brassica* species possess potent anticancer properties, mainly attributable to glucosinolates, or rather to their derivatives isothiocyanates and indoles [46]. In addition, flavonoids and other phenolic compounds were reported to be effective anticancer agents, acting through numerous mechanisms of action including destabilization of the oxidative state of cancer cells [47]. The potential antiproliferative effect of *B. drepanensis* total extract was tested by MTT assay on four tumour cell lines, namely DU145 (prostatic cancer), CaCo-2 (colon carcinoma), 1321N1 (astrocytoma), and U87 MG (glioblastoma). The exposure of these cell lines to different concentrations of the extract for 24 h resulted in an antiproliferative effect only in the CaCo-2 cell line, with a slight decrease in cell proliferation at concentrations above 250 μg/mL (Figure 3D) accompanied by an increment in LDH release (Figure 7). The increase in the release of LDH is a well-known index of necrotic cell death and cytotoxicity. Considering the good antioxidant properties previously evidenced in the inflammatory model, we investigated ROS production in CaCo-2 cells. At the cytotoxic concentrations, the extract counteracted the production of ROS in a dose-dependent manner (Figure 4B). These results suggest that the antiproliferative and cytotoxic activity of the phytocomplex could be related to the alteration of the redox state and consequent stimulation or suppression of some signalling pathways [48]. Certain polyphenols, including kaempferol, have been shown to regulate several signalling pathways involved in diverse cancer stages [49].

## 4. Materials and Methods

### 4.1. Chemicals, Apparatus, and Reagents

Porcine pancreatic lipase, α-amylase from porcine pancreas, α-glucosidase from *Saccharomyces cerevisiae*, Tween 20, sulfuric acid, sodium carbonate, 2,2-diphenyl-1-picrylhydrazyl (DPPH), superoxide dismutase (SOD), tripyridyltriazine (TPTZ), 2,2′-azino-bis(3-ethylbenzothiazoline-6-sulfonic) acid (ABTS) solution, β-carotene, linoleic acid, ascorbic acid, propyl gallate, orlistat, butylated hydroxytoluene (BHT), 4-nitrophenyl octanoate (NPC), maltose, *o*-dianisidine dihydrochloride, 4-nitrophenyl octanoate, and peroxidase/glucose oxidase (PGO) were purchased from Sigma-Aldrich S.p.a. (Milan, Italy). Acarbose was purchased from Serva (Heidelberg, Germany). Analytic-grade organic solvents were purchased from VWR (Milan, Italy). UHPLC-grade water (18 mΩ) was prepared using a Mill-Q purification system (Millipore Co., Bedford, MA, USA). UHPLC-grade MeOH, formic acid, and H_2_O were purchased from Romil-Deltek (Pozzuoli, Italy).

One-dimensional and two-dimensional NMR experiments were recorded at 300 K in CD_3_OD on a Bruker DRX-400 spectrometer (Bruker BioSpin GmBH, Milan, Italy). ESI-MS/MS experiments (direct injections) were performed on a LCQ Advantage ion trap mass spectrometer (flow rate 5 µL/min) equipped with Xcalibur software (ThermoFinnigan, San Jose, CA, USA). Gel filtration chromatography was performed over Sephadex LH-20 using a column equipped with a peristaltic pump (Pharmacia, Uppsala, Sweden) and eluting with MeOH:H_2_O (50% *v/v*), flow rate 1.0 mL/min, collection volume 5 mL. Flash column chromatography was conducted on a Biotage^®^ Isolera (Agilent, Milan, Italy) flash purification system (flash silica gel 60, SNAP cartridge 50 g, flow rate 40 mL/min, collection volume 27 mL) eluting with solvent mixtures at increasing polarity (*n*-hexane, CHCl_3_, and MeOH). RP-HPLC separations were carried out on a C-18 μ-Bondapak column, 30 cm × 7.8 mm, 10 μm particle size (Waters Corporation, Milford, MA, USA), using mixtures of MeOH and H_2_O as eluents, flow rate 2.0 mL/min. The HPLC equipment was composed of a Shimadzu LC-8A series pumping system, a Shimadzu RID-10A refractive index detector, and a Shimadzu injector (Shimadzu Corporation, Kyoto, Japan). Thin-layer chromatography (TLC) was conducted on silica gel 60 F_254_ (0.20 mm thickness) plates (Merck, Darmstadt, Germany) using *n*-BuOH:CHCl_3_:H_2_O (60:15:25) as an eluent, as well as mixtures of chloroform and methanol, and Ce(SO4)_2_/H_2_SO_4_ as a spray reagent (Sigma-Aldrich, Milano, Italy). UHPLC-mass spectrometry (MS) analysis was performed on a system composed of a Vanquish Flex LC binary pump and a Q Exactive Plus high-resolution MS, Orbitrap-based FT-MS system (Thermo Fischer Scientific Inc., Dreieich, Germany) equipped with an ESI source. The sample (R_w_) was dissolved in MeOH at a concentration of 2 mg/mL, then centrifuged. The supernatant was injected (5 μL) into a C-18 Kinetex^®^ Biphenyl column (100 × 2.1 mm, 2.6 μm particle size) equipped with a Security Guard^TM^ Ultra cartridge (Phenomenex, Bologna, Italy). The elution was performed using formic acid in MeOH 0.1% *v/v* (solvent A) and formic acid in H_2_O 0.1% *v/v* (solvent B) at a flow rate of 0.5 mL/min and developing a linear solvent gradient from 5 to 45% A in 10 min. The temperatures of the autosampler and column oven were maintained at 4 and 35 °C, respectively. ESI-MS spectra were acquired in a scan range of 150–1200 *m/z* in negative and positive ion ESI mode, operating in full MS/MS scan (70,000 resolution, maximum injection time 220 ms) and data-dependent (17,500 resolution, maximum injection time 60 ms). Ionization parameters were used as previously reported [50]. Xcalibur 3.1 software (Thermo Scientific, San Jose, CA, USA) was used for data elaboration.

### 4.2. Plant Collection and Extraction Procedure

*B. drepanensis* was collected in the locus classicus of the taxon, in the area of Mount Erice (Trapani, Sicily, Italy) (38°02′07.04″ N 12°35′31.7″ E) in May 2019. The specimen was obtained and authenticated by botanist Prof. F.M. Raimondo. A voucher specimen of the plant (No. 05/19) was deposited in the herbarium of the Department of Drug and Health Sciences, Section of Biochemistry.

After harvesting, the leaves (980 g) were dried in an oven at 40 °C for 24 h, obtaining 400 g of dried plant material. Dry leaves were extracted at 40 °C in 70% ethanol for 4 h. The extraction was repeated three times. The extract solution was then filtered and evaporated to dryness under reduced pressure with a rotatory evaporator, obtaining about 23.0 g of dry extract.

### 4.3. Determination of Total Phenolic Content

The TPC of the leaf extract was determined spectrophotometrically by the Folin–Ciocalteau method, as reported by Acquaviva et al. [51]. The TPC value was compared to a calibration curve of a known amount of gallic acid and expressed as mg of gallic acid equivalent (GAE/g extract). The TFC was evaluated spectrophotometrically, compared to a calibration curve of a known amount of catechin, and expressed as mg of catechin equivalent (CE/g extract). The data were obtained from three independent determinations.

### 4.4. Isolation of Pure Compounds

The hydroalcoholic extract (10.9 g) was first partitioned between ethyl acetate/H_2_O, and then the aqueous phase was extracted with *n*-BuOH. All extract solutions were dried under vacuum, finally obtaining 0.73, 0.30, and 8.57 g of EtOAc, *n*-BuOH, and aqueous extracts, respectively. The *n*-BuOH extract (304.0 mg) was purified by RP-HPLC eluting with MeOH-H_2_O (25% *v*/*v*) to obtain pure compounds **1** (*t*_R_ = 9.2 min, 1.0 mg), **2** (*t*_R_ = 10.8 min, 0.3 mg), **3** (*t*_R_ = 26.2 min, 1.5 mg), **4** (*t*_R_ = 28.5 min, 1.8 mg), and **5** (*t*_R_ = 33.1 min, 1.9 mg). The aqueous residue (2.8 g) was submitted to a Sephadex LH-20 column, using MeOH-H_2_O (50% *v*/*v*) as an eluent, to yield 4 major fractions based on the TLC profile (A-D). Fractions B and C were purified by RP-HPLC eluting with MeOH-H_2_O (20% *v/v*) to obtain pure compounds **1** (*t*_R_ = 10.3 min, 1.2 mg) from B, previously isolated from the *n*-BuOH extract, and compounds **6** (*t*_R_ = 6.9 min, 1.7 mg) and **7** (*t*_R_ = 54.8 min, 7.0 mg) from C. Fraction D was further purified on RP-HPLC eluting with MeOH-H_2_O (35% *v*/*v*), obtaining pure compounds **8** (*t*_R_ = 11.2 min, 0.1 mg) and **2** (*t*_R_ = 14.8 min, 0.9 mg). The EtOAc extract (545.0 mg) was subjected to flash silica gel column chromatography using a Biotage^®^ system and collecting 4 major fractions that were grouped based on the TLC results. The purification by RP-HPLC of obtained fractions failed in isolating secondary metabolites of interest. All isolated compounds were identified by 1D and 2D-NMR experiments as well as ESI-MS.

### 4.5. SOD-like Activity

The scavenger effect of *B. drepanensis* extract and the fractions on superoxide anions was recorded as a decrease in absorbance at λ = 340 nm, as previously reported by Salerno et al. [52]. Results are reported as a mean of 50% inhibitory concentration (IC_50_) of NADH oxidation. SOD (80 mU) was used as a reference compound.

### 4.6. DPPH Test

Free radical-scavenging capacity was analysed through the ability of the total extract and the fractions to bleach the stable radical DPPH. The results were compared to Trolox (30 µM), a water-soluble derivative of vitamin E used as a reference compound. Briefly, after 10 min at room temperature, the absorbance at λ = 517 nm of the DPPH reaction mixture containing different concentrations of *B. drepanensis* extract (125–1000 µg/mL) in 1 mL of ethanol was recorded [52]. Results, expressed as decrease in absorbance, represent the average ± S.D. of three independent experiments.

### 4.7. ABTS Assay

In this assay, a solution of ABTS radical cations was mixed with a potassium persulphate solution and stored at room temperature for 12 h. The obtained solution was diluted with ethanol, read to obtain an absorbance of 0.70 at 734 nm, and added to the tested sample (1–400 μg/mL). After 6 min, the absorbance was read at 734 nm. Ascorbic acid was the positive control. The ABTS radical-scavenging ability was calculated with this equation: [(A0−A1)/A0] × 100, where A0 is the absorbance of the control and A1 is the absorbance in the presence of the samples.

### 4.8. Ferric Reducing Activity Power (FRAP) Assay

The FRAP test was used to measure antioxidant power based on the reduction at low pH of ferric-tripyridyltriazine (Fe^3+^-TPTZ) to a blue-coloured ferrous-tripyridyltriazine complex (Fe^2+^-TPTZ) [53]. The FRAP reagent was prepared by mixing a tripyridyltriazine (TPTZ) solution, acetate buffer FeCl_3_, and HCl, and was added to the tubes containing the sample at a concentration of 2.5 mg/mL. The absorbance was measured at 595 nm after 30 min of incubation at room temperature. Butylated hydroxytoluene (BHT) was used as the positive control, and results have been expressed as μMFe (II)/g of extract.

### 4.9. Carotene Bleaching Assay

The β-carotene bleaching test was performed following the procedure previously described [53], using propyl gallate as the positive control. A solution of linoleic acid, Tween 20, and β-carotene was prepared and added to a 96-well microplate containing samples in concentrations in the range 2.5–100 μg/mL. The microplate was located in a water bath at 45 °C and the absorbance was read at 470 nm against a blank at t = 0 and after 30 and 60 min of incubation.

### 4.10. Cell Culture and Treatments

The human foreskin fibroblast cell line HFF-1 (ATCC^®^ SCRC-1041.1) was maintained in DMEM supplemented with 15% *v/v* foetal bovine serum (FBS), 4.5 g/L glucose, 100 U/mL penicillin, and 100 mg/mL streptomycin. HFF-1 was used as an in vitro human model for preliminary toxicity screening. Mouse leukemic monocyte-macrophage cells RAW 264.7 (Sigma-Aldrich 91062702) were cultured in EMEM supplemented with 2 mM L-Glutamine (Euroclone, Milan, Italy), 1% non-essential amino acids, 10% FBS, and penicillin/streptomycin/amphotericin. RAW 264.2 was used as an in vitro model of LPS-induced inflammation. Human colon carcinoma cells CaCo-2 (ATCC^®^, HTB-37) were cultured in DMEM supplemented with 10% FBS, 1 mmol/L sodium pyruvate, 2 mmol/L l-glutamine, streptomycin (50 mg/mL), and penicillin (50 U/mL). Prostate cancer DU145 cells (ATCC^®^ HTB-81) were cultured in RPMI supplemented with 5% (*v/v*) FBS, 100 units/mL of penicillin, and 100 mg/mL of streptomycin. Human 1321N1 astrocytoma (Sigma-Aldrich, 86030402) and U87 MG glioblastoma cells (ATCC^®^ HTB-14) were grown in Dulbecco’s modified Eagle’s medium (DMEM) supplemented with 5% (*v/v*) FBS, 100 units/mL of penicillin, and 100 mg/mL of streptomycin. Cancer cells were used as in vitro preliminary screening of anticancer activity. All cell lines at sub-confluent conditions were plated at a constant density (8 × 10^3^ cells/well) to obtain identical experimental conditions in the different tests and to achieve a high accuracy of the measurements.

### 4.11. MTT Assay

HFF-1, RAW 264.7, CaCo-2, DU145, 1321N1, and U87 MG cells were treated with different concentrations of total extract (25–50–100–150–250–500–1000 μg/mL), while RAW 264.7 cells were also treated with the fractions obtained from the total extract (EtOAc, *n*-BuOH, and H_2_O) at the same concentrations (25–50–100–150–250–500–1000 μg/mL) for 24 h. Doxorubicin was used as a standard of cytotoxicity for the CaCo-2 cells. The MTT test determines cell viability because it measures the conversion of tetrazolium salts to yield coloured formazan in the presence of metabolic activity. The amount of formazan is proportional to the number of living cells. The absorbance of the converted formazan was measured using a microplate spectrophotometer reader (Titertek Multiskan, Flow Laboratories, Helsinki, Finland) at λ = 570 nm. The results were presented as the percent of control data [38].

### 4.12. Reactive Oxygen Species Assay

The fluorescent probe 2′,7′-dichlorofluorescein diacetate (DCFH-DA) was used to evaluate ROS levels [38]. The fluorescence (corresponding to the oxidized radical species 2′,7′-dichlorofluorescein, DCF) was monitored spectrofluorometrically (excitation, λ = 488 nm; emission, λ = 525 nm). The total protein content was evaluated for each sample and the results were reported as percentage of fluorescence intensity/mg protein respect to control. Protein content was determined using a Sinergy HTBiotech instrument by measuring the absorbance difference at λ = 280 and λ = 260 nm.

### 4.13. Total Thiol Group Determination

Non-proteic total thiol groups were measured in 200 μL of lysate supernatant as previously described [47]. The assay is based on the reaction of thiols with 2,2-dithio-*bis*-nitrobenzoic acid. The spectrophotometric measures were conducted at λ = 412 nm with Sinergy HT Biotech instrument. Results are expressed in nmol/mg protein.

### 4.14. Measurement of NO· Release

The inhibitory effect of total extract and the fractions on NO· production was defined by measuring nitrite levels with Griess reagent, as reported by Calabrese et al. [41]. RAW 264.7 cells were pre-treated with different concentrations of the total extract and the fractions for 24 h and successively stimulated with LPS (2 μg/mL) for 2 h. At the end of the treatments, the culture medium (250 μL) was mixed with 250 μL of Griess reagent and incubated at room temperature for 10 min according to manufacturer instructions. The assay is based on the reaction of diazocopulation of nitrite with the Griess reagent. The nitrite content in culture media was determined at 540 nm using a Synergy HT plate reader (BioTek Instruments, Inc., Winooski, VT, USA). Results were calculated by comparison with OD550 of standard solutions of sodium nitrite prepared in H_2_O and expressed as percentage of nitrite production with respect to untreated and LPS stimulated cells.

### 4.15. Pancreatic Lipase Inhibitory Activity Test

Pancreatic lipase inhibitory activity was evaluated as previously described [54]. In brief, an aqueous solution of lipase (1 mg/mL), a solution of 4-nitrophenyl octanoate (5 mM in dimethyl sulfoxide), and Tris-HCl buffer (pH 8.5) were prepared. These solutions were mixed to the sample (at concentrations in a range from 2.5 to 40.0 mg/mL) and incubated for 30 min at 37 °C. Then, the absorbance was read at 405 nm. Orlistat was used as the positive control.

### 4.16. Amylase Inhibitory Activity Test

In the α-amylase inhibitory test, the α-amylase solution was prepared by dissolving 25.3 mg of the enzyme in 100 mL of cold distilled water following the previously described procedure [55]. The colorimetric reagent was prepared by mixing a sodium potassium tartrate solution and 96 mM 3,5-dinitrosalicylic acid solution. Different concentrations of *B. drepanensis* extract (in a range from 12.50 to 1000 μg/mL) were added to the starch solution and left to react with the enzyme at room temperature. The absorbance was read at 540 nm. Acarbose was used as a positive control.

### 4.17. α-Glucosidase Inhibitory Activity Test

This test was performed as previously reported [55]. In brief, *B. drepanensis* extract (concentrations ranging from 12.50 to 1000 μg/mL) was stirred into a maltose solution previously prepared at 37 °C. The reaction was started by adding the enzyme and was stopped after 30 min of incubation at 37 °C by adding a perchloric acid solution. The supernatant was mixed with *o*-dianisidine (DIAN) colour reagent solution and peroxidase–glucose oxidase (PGO) system colour reagent solution and was incubated for 30 min at 37 °C. The absorbance was measured at 540 nm. Acarbose was used as a positive control.

### 4.18. LDH Assay

Lactic dehydrogenase (LDH) release was measured spectrophotometrically in the culture medium and in the cellular lysates at λ = 340 nm by analysing NADH reduction [47]. The percentage of LDH release was calculated as percentage of the total amount, considered as the sum of the enzymatic activity present in the cellular lysate and in culture medium. This assay evaluates cell necrosis as a result of cell membrane disruption. Results are expressed as percentage in LDH released.

### 4.19. Statistical Analysis

The obtained experimental results are reported as the mean ± standard deviation (SD). Statistical analysis of the data was carried out by one-way analysis of variance (ANOVA), followed by the Tukey’ post-hoc test using Graph Prism version 5. Differences were considered significant when *p* ≤ 0.05.

## 5. Conclusions

*B. drepanensis* represents a safe source of bioactive compounds. The above results revealed that thanks to the rich phytocomplex, higher than that of the most-cultivated and -consumed varieties of *B. oleracea*, the extract showed marked primary antioxidant properties and significant activity in positively modulating the early stages of oxidative stress and inflammation by preserving glutathione content and inhibiting ROS and NO production in RAW 264.7 cells. Although the extract evidenced a weak activity on α-amylase and α-glucosidase, a significant inhibitory effect on pancreatic lipase activity was found. These data confirm the key role of plant secondary metabolites in preventing and ameliorating alteration of metabolism parameters typical of metabolic syndrome. Among the cancer cell lines tested for cytotoxic potential, the extract showed antiproliferative effects and cytotoxicity only against CaCo-2 cells. Furthermore, the necrotic cell death detected was probably related to the dysregulation of the redox environment as suggested by the decrease in ROS production. Researching new dietary ingredients with remarkable healthy biological activities, without side effects, for the prevention of disorders related to lifestyle seems to be a topic that will further stimulate research in the field of wild plants.

Biodiversity is an important factor involved in many aspects of human life, including health and wellbeing. Wild plant species and subspecies, with their unique characteristics, may represent a useful tool for the economy of the territory for both their possible sustainable cultivation and marketing as superfoods (since some are traditionally known as edible plants) and for their use in nutraceutical or botanical applications.

## Figures and Tables

**Table 1:** Cell free antioxidant activities.

*B. drepanensis* Extract	ABTS Test IC_50_ (μg/mL)	FRAP Test IC_50_ (μMfe (II)/g)	DPPH Test IC_50_ (μg/mL)	SOD-like Activity IC_50_ (μg/mL)	β-Carotene Bleaching Test IC_50_ (μg/mL)	
EtOH-H_2_O	3.32 ± 0.88 *	10.46 ± 1.17 *	368 ± 1.7 *	66.66 ± 1.1 *	38.34 ± 2.51 *	47.95 ± 2.64 *
*n*-BuOH	2.5 ± 1.1 *	7.09 ± 1.8 *	301 ± 2.3 *	40.03 ± 1.2 *	29.04 ± 3.1 *	35.60 ± 1.4 *
EtOAc	>50	>150	>700	>200	N.D.	N.D.
H_2_O	>50	>150	>700	>200	N.D.	N.D.
Positive control						
Ascorbic acid	1.73 ± 0.06					
BHT		63.24 ± 2.34				
Trolox			15 μM ± 0.62			
SOD				40 mU ± 0.85		
Propyl gallate					0.09 ± 0.04	0.09 ± 0.04

Values are the mean ± S.D. of three experiments in triplicate. Confidence intervals calculated by two-way ANOVA: * *p* < 0.005 compared with the positive controls.

**Table 2:** Hypoglycaemic and hypolipidemic activity (IC_50_ value, μg/mL) of *B. drepanensis*.

Sample	α-Amylase	α-Glucosidase	Lipase
*B. drepanensis* whole extract	504.25 ± 5.11 *	364.15 ± 3.14 *	74.84 ± 3.67 *
Positive control			
Acarbose	35.52 ± 0.93	50.14 ± 1.31	
Orlistat			37.45 ± 1.08

Values are the mean ± SD of three experiments in triplicate. Confidence intervals calculated by two-way ANOVA: * *p* < 0.005 compared with the positive controls.

**Table 3:** Total phenol and flavonoid content of *B. drepanensis* whole extract.

*B. drepanensis* Whole Extract	Total Phenol Content (TPC) mg GAE/g Extract	Total Flavonoid Content (TFC) mg CE/g Extract
	29.87 ± 0.14 *	26.12 ± 0.09 *

Values, expressed as mg gallic acid (GAE) and catechin (CE) equivalents, are the mean ± S.D. of three experiments in triplicate. Confidence intervals calculated by two-way ANOVA: * *p* < 0.005.

## Data Availability

The data presented in this study are available on request from the corresponding author.
